# Generation of Red Blood Cells from Human Pluripotent Stem Cells—An Update

**DOI:** 10.3390/cells12111554

**Published:** 2023-06-05

**Authors:** Shin-Jeong Lee, Cholomi Jung, Jee Eun Oh, Sangsung Kim, Sangho Lee, Ji Yoon Lee, Young-sup Yoon

**Affiliations:** 1 Severance Biomedical Science Institute, Yonsei University College of Medicine, Seoul 03722, Republic of Korea; shinjeonglee@yuhs.ac (S.-J.L.); cjung90@yuhs.ac (C.J.); jeeeunoh@yuhs.ac (J.E.O.); skim472@yuhs.ac (S.K.); 2 Research and Development Center, KarisBio Inc., 50-1 Yonsei-Ro, Avison Biomedical Research Center Room 525, Seodaemun-gu, Seoul 03722, Republic of Korea; 3 Department of Internal Medicine, Graduate School of Medical Science, Brain Korea 21 Project, Yonsei University College of Medicine, Seoul 03722, Republic of Korea; 4 Division of Cardiology, Department of Medicine, Emory University School of Medicine, Atlanta, GA 30322, USA; sangho.lee@emory.edu

**Keywords:** human pluripotent stem cell, erythroid cell, red blood cell, hematopoietic stem cell, primitive erythropoiesis, definitive erythropoiesis, enucleation

## Abstract

Red blood cell (RBC) transfusion is a lifesaving medical procedure that can treat patients with anemia and hemoglobin disorders. However, the shortage of blood supply and risks of transfusion-transmitted infection and immune incompatibility present a challenge for transfusion. The in vitro generation of RBCs or erythrocytes holds great promise for transfusion medicine and novel cell-based therapies. While hematopoietic stem cells and progenitors derived from peripheral blood, cord blood, and bone marrow can give rise to erythrocytes, the use of human pluripotent stem cells (hPSCs) has also provided an important opportunity to obtain erythrocytes. These hPSCs include both human embryonic stem cells (hESCs) and human induced pluripotent stem cells (hiPSCs). As hESCs carry ethical and political controversies, hiPSCs can be a more universal source for RBC generation. In this review, we first discuss the key concepts and mechanisms of erythropoiesis. Thereafter, we summarize different methodologies to differentiate hPSCs into erythrocytes with an emphasis on the key features of human definitive erythroid lineage cells. Finally, we address the current limitations and future directions of clinical applications using hiPSC-derived erythrocytes.

## 1. Introduction

Red blood cell (RBC) transfusion is crucial not only for patients suffering from RBC inefficiency, including anemia and hemoglobin disorders, but also for modern medical practices, such as surgeries and cancer treatments [1]. However, blood donor shortages and risks associated with transfusions remain a key challenge. The in vitro generation of RBCs or erythrocytes has been proposed as a viable alternative. Several studies used hematopoietic stem cells and/or progenitor cells (HSCs and/or HSPCs) from adult and cord blood to derive RBCs [2,3]. However, adult- and cord blood-derived HSCs and/or HSPCs are a limited source, and adult- and cord blood-derived RBCs are unsustainable.

Human pluripotent stem cells (hPSCs), including embryonic stem cells (ESCs) and induced pluripotent stem cells (iPSCs), offer hope for an unlimited supply of RBCs, owing to their self-renewal capacity [4,5,6]. The derivation of RBCs from hPSCs recapitulates the defining events of erythropoiesis, a developmental process in which HSCs and/or HSPCs are committed to the erythroid lineage cells [7]. Accordingly, many have attempted to develop stepwise protocols that first differentiate hPSCs into HSCs and/or HSPCs, into erythroid progenitor cells, and into erythrocytes [8,9]. These steps are regulated by providing a suitable microenvironment or niche and sequential treatments of growth factors, cytokines, and small molecules pertaining to the cellular and molecular mechanisms underlying the process of erythropoiesis [10]. While the resulting hPSC-derived RBCs exhibit certain characteristics of human erythrocytes, much remains to be done to effectively assess their therapeutic potential at the clinical level.

**Figure 1:**
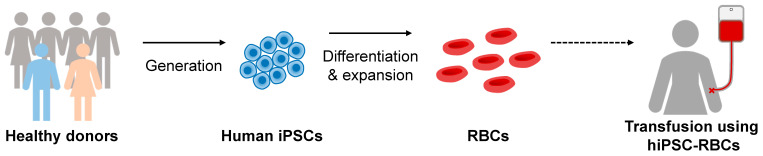
Proposed scheme for generation of clinically suitable hiPSC-derived RBCs for transfusion. Human iPSCs can be generated from somatic cells, such as skin fibroblasts and blood cells. Human iPSCs can then be differentiated into RBCs. After expansion, the resulting RBCs can be used for transfusion. Solid arrow indicates a verified and validated step. Dashed arrow indicates a step yet to be determined.

In this review, we first describe the developmental biology of erythropoiesis and cellular and molecular mechanisms regulating its process. Thereafter, we discuss protocols for the generation of erythroid lineage cells from hPSCs. In particular, we list different combinations of growth factors and cytokines for each culture system and assessments for the molecular and functional characterization of hPSC-derived RBCs. We then highlight the key features that hPSC-derived RBCs should possess prior to their clinical translation. Lastly, we discuss the current limitations and future directions of hPSC-derived RBCs for potential clinical implementations (Figure 1).

## 2. Erythropoiesis

**Figure 2:**
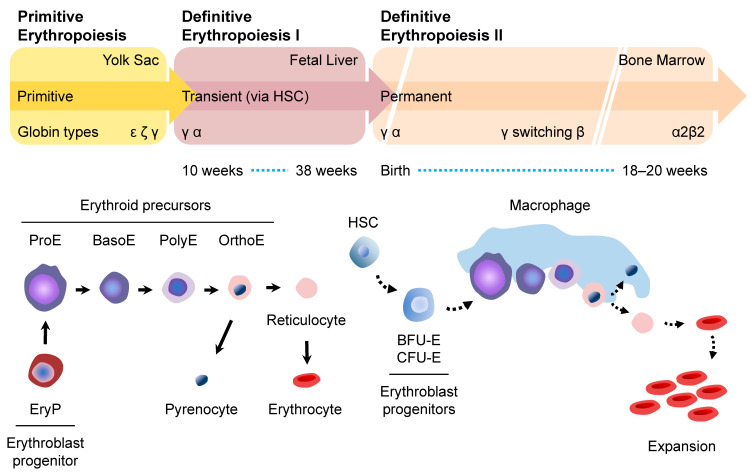
Overview of primitive and definitive erythropoiesis. Primitive erythropoiesis occurs within the yolk sac, while definitive erythropoiesis occurs in the fetal liver and postnatal BM. The transition to erythrocytes is identical in primitive and definitive erythropoiesis. Erythroid progenitors are EryP in primitive erythropoiesis, while BFU-E and CFU-E cells are definitive erythroid progenitors. Erythroblasts include ProE, BasoE, PolyE, and OrthoE. After enucleation, OrthoE becomes either pyrenocyte or reticulocyte. While pyrenocytes are often engulfed and removed by macrophages, reticulocytes further mature into erythrocytes or RBCs.

Erythropoiesis is the process of generating RBCs in the bone marrow (BM). Like hematopoiesis, erythropoiesis occurs in two sequential stages or “waves”, which are dependent on the timing and anatomical sites [11,12]. They are primitive erythropoiesis and definitive erythropoiesis (Figure 2).

**Figure 3:**
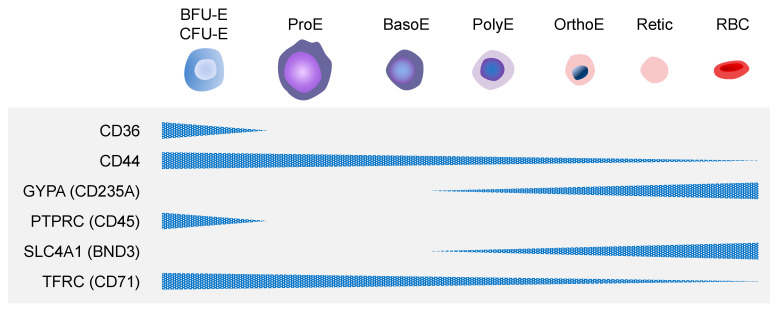
Key markers of the erythroid lineage cells. Surface markers that define the erythroid lineage cells are listed. In general, BFU-E and CFU-E cells express CD36, CD44, PTPRC, and TFRC. Erythroblasts, including reticulocytes express CD44, GYPA, SLC4A1, and TFRC. Erythrocytes or RBCs express GYPA and SLC4A1. The expression levels are relative.

Primitive erythropoiesis refers to the transient presence of RBCs within the yolk sac, while definitive erythropoiesis refers to the presence of RBCs in the fetal liver and postnatal BM [11]. In both primitive and definitive erythropoiesis, the status of erythrocytes is transitioned through three compartments: erythroid progenitor cell, erythroblast precursor cell, and RBCs. In primitive erythropoiesis, primitive erythroid (EryP) progenitors transiently appear and give rise to erythroid precursor cells, while in definitive erythropoiesis, definitive erythroid progenitors that include burst-forming unit erythroid (BFU-E) cells and colony-forming unit erythroid (CFU-E) cells, give rise to erythroid precursor cells. These precursor cells include proerythroblasts (ProEs), basophilic erythroblasts (BasoEs), polychromatophilic erythroblasts (PolyEs), and orthochromatic erythroblasts (OrthoEs). In general, erythroid precursor cells are erythroblasts as well as reticulocytes, and enucleated RBCs are classified as erythrocytes. The erythroid lineage cells represent erythroblasts to erythrocytes. These cells express transferrin receptor (TFRC; CD71), solute carrier family 4 member 1 (SLC4A1; BND3), and glycophorin A (GYPA; CD235A) [13] (Figure 3). In addition to these conventional RBC markers, CD36 and CD44 have been also used to distinguish erythroblasts from TFRC^+^ cells in different developmental stages [14]. In the case of hPSC-derived erythroid lineage cells, sialophorin (SPN; CD43) and protein tyrosine phosphatase receptor type C (PTPRC; CD45) are used as markers for identifying the committed hematopoietic lineage cells from hPSCs.

### 2.1. Primitive Erythropoiesis

Primitive erythropoiesis originates in the yolk sac. Specifically, the primitive erythroid cells first appear in “blood islands” and complete their maturation into erythrocytes in the bloodstream [15]. These blood islands arise from mesodermal cells between embryonic days 7 and 7.5, and quickly give rise to a cluster of inner blood cells surrounded by an outer endothelial lining by E9.5 [16]. Primitive erythroblasts then undergo progressive maturation towards primitive RBCs, characterized by erythroblast expansion and proliferation, fetal globin accumulation, and decreased cell size. Furthermore, primitive RBCs are not fully mature cells because they possess nuclei and are enriched with ε- and γ-globins rather than β-globin [17,18]. Interestingly, these erythroblasts also lose vimentin, intermediate filaments, and microtubules prior to entering the bloodstream [19,20].

### 2.2. Definitive Erythropoiesis

Definitive erythropoiesis, on the other hand, occurs later in the developmental stage and continues throughout the postnatal life. It is driven by HSCs and/or HSPCs in the fetal liver and postnatal BM [11]. Like primitive erythroid cells from primitive erythropoiesis, HSCs and/or HSPCs undergo the transition to definitive erythrocytes. First, HSCs and BFU-E and CFU-E cells are first found and transiently expand their cell numbers in the fetal liver [21,22,23,24,25]. These cells then become erythroblasts transitioning from ProE, to BasoE, to PolyE, to OrthoE, and ultimately to reticulocytes. Definitive erythroblasts exhibit progressive characteristics similar to their primitive counterparts. However, these RBCs in the fetal liver are not functional due to the non-conversion of γ-globin to β-globin. Cellular maturation next occurs in the BM during the stages of postnatal life. Definitive RBCs derived from definitive erythropoiesis can function physiologically to transport oxygen, with a lifespan of approximately 120 days [26]. These definitive RBCs are essential for clinical application in transfusion medicine.

## 3. Mechanisms of Erythropoiesis

### 3.1. Paracrine Mechanisms of Erythropoiesis

There are several cytokines and growth factors involved in and critical for erythropoiesis. Among them, erythropoietin (EPO), which is mainly produced in the kidney, is the most well-known and studied cytokine. Earlier studies revealed that EPO expression was induced by hypoxia-inducible factor (HIF), and in erythroid progenitor cells, such as BFU-E and CFU-E cells, EPO and its receptor (EPOR) trigger canonical Janus kinase 2/signal transducer and activator of transcription 5 (JAK2/STAT5), rat sarcoma/mitogen-activated protein kinase/extracellular signal-regulated kinase (RAS/MEK/ERK), and phosphatidylinositol 3-kinase (PI3K) pathways [27]. Several studies suggested that EPO signaling is expendable for the development and specification of primitive erythroid progenitor cells in the yolk sac, although it is required for the survival of erythroid progenitor cells in in vitro cell culture [28,29]. However, EPO signaling is essential for definitive erythropoiesis by promoting the survival, proliferation, and apposite timing of the terminal maturation of primitive erythroid progenitor cells [28,30]. Because EPO instead of iron is required for cell proliferation in the steps up to BasoE, the kidney immediately produces EPO to deliver to BM, when erythrocytes have low iron levels. Stem cell factor (SCF) is another well-known growth factor and a ligand of KIT proto-oncogene, receptor tyrosine kinase (KIT). The failure of SCF signaling in c-kit mutant mice reduced the number of mature CFU-E cells in the fetal liver, whereas the number of BFU-E cells was maintained, suggesting that SCF promotes erythropoiesis [31]. Furthermore, another study suggested that SCF rapidly induced tyrosine phosphorylation of the EPOR through KIT, which was physically associated with the cytoplasmic domain of EPOR. Thus, SCF activates EPOR via KIT to induce the proliferation and maturation of CFU-E cells [32]. Additionally, the synergistic regulation by SCF together with IL3 and EPO has been demonstrated in early erythropoiesis [33,34]. In addition to SCF, insulin, insulin-like growth factor (IGF), activin A, and angiotensin II also positively regulate erythropoiesis, whereas transforming growth factor-β (TGFβ) signaling, growth differentiation factor 11 (GDF11; BMP11), γ-interferon, and tumor necrosis factor-α (TNFα) inhibit terminal erythroid maturation in vitro, although their roles in in vivo erythropoiesis remain to be confirmed [35,36].

### 3.2. Erythroblastic Islands

Cellular interaction within erythroblastic islands (EBIs) is pivotal to mature the erythroid lineage cells. In mammals, EBIs represent a specialized niche in which erythroid precursor cells proliferate, differentiate, and enucleate to become functional RBCs. These EBIs are mostly found in BM during steady-state erythropoiesis but extend to the fetal liver and adult spleen for erythropoiesis in stress conditions [37,38,39,40]. For example, in response to inflammation and anemia, this extension of EBIs to the fetal liver and adult spleen accelerates erythrocyte production. During the differentiation of erythroid progenitor cells, the process of deriving nucleated precursor cells from ProE to OrthoE occurs within EBIs [39]. These EBIs consist of a specialized macrophage on the center encompassed by developing erythroblasts [39,41]. Cell-to-cell contact in EBIs is critical for erythroblast differentiation. The communications between central macrophages and erythroblasts orchestrate proliferation and differentiation of various erythroblasts [14,42]. Central macrophages not only provide molecular cues for differentiation and proliferation but also clear out discharged nucleases and organelles by phagocytosis during enucleation of OrthoE and during terminal differentiation from reticulocytes into erythrocytes [43,44].

### 3.3. Molecular Mechanisms of Erythropoiesis

**Figure 4:**
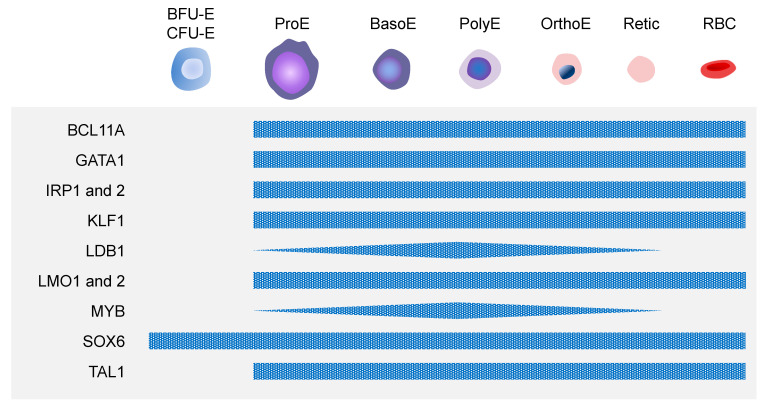
Key transcription factors involved in erythropoiesis. Transcription factors that play important roles in erythropoiesis.

In erythroid differentiation, core transcriptional networks play crucial roles, which include gata binding protein 1 (GATA1), T-cell acute lymphoblastic leukemia/stem cell leukemia (TAL1/SCL), Krüppel-Like Factor 1 (KLF1), LIM domain only 2 (LMO2), and LIM domain binding protein 1 (LDB1) (Figure 4).

DNA-binding transcription factors, such as GATA1, TAL1, and KLF1, are the master regulators that cooperatively bind to active enhancers, form core complexes with LMO1 and 2 and LDB1 proteins, and impart their regulatory functions by the dynamic recruitment of additional transcription cofactors in a lineage- and stage-specific manner [45]. For example, the GATA1/TAL1/LMO2/LDB1 complex recruits stage-specific coregulators, such as iron responsive element 2 and 6 (IRF2 and 6) and myeloblastosis oncogene (MYB), and controls the differential expression of erythroid genes for the adult-stage transcriptional program [46]. The importance of such transcription factors of the core network can be found in human diseases. Diamond-Blackfan anemia (DBA) is a hypoplastic anemia characterized by the impaired production of RBCs, and some of the pathogenic mutations were mapped to a splice site of the GATA1 gene, causing the impairment of full-length protein production [47]. Furthermore, the mutations in the KLF1 gene result in multiple anemia phenotypes and sometimes elevated levels of fetal hemoglobin (HbF) [48]. In addition, a knockout of these transcription factors—GATA1 and KLF1—causes severe defects in erythropoiesis and lethality before gestation [49,50,51,52]. Interestingly, Spi-1 proto-oncogene (SPI1; PU.1), a master regulator of myeloid-lymphoid differentiation, represses the expression of the core transcriptional network genes, including GATA1 cofactors, and negatively regulates terminal erythroid differentiation. Conversely, GATA1 binds to and represses many SPI1 target genes in erythroid progenitors [53]. Besides core transcription factors, other transcriptional cofactors also play important roles in erythropoiesis. BCL11 transcription factor A (BCL11A) is a key regulator of the hemoglobin switch from a fetal to an adult form, silencing HbF expression. Sankaran et al. demonstrated that the full-length forms of BCL11A are almost exclusively in adult-stage erythroblasts, and that BCL11A binds to the nucleosome remodeling and histone deacetylase (NuRD)–repressive complex; and, to GATA1 and a GATA1 cofactor, zinc finger protein, FOG family member 1 (ZFPM1; FOG1) in erythroid cells [54]. Another transcription factor, MYB, has been known to be a key regulator of hematopoiesis and erythropoiesis and a negative regulator of HbF levels. In a recent study, MYB is specifically expressed only in erythroid cells and its expression is modulated through the binding of transcriptional complex of core factors, such as GATA1, TAL1, LDB1, and KLF1, to a long-range enhancer of MYB [55]. Additionally, Cantu et al. showed that SRY-box transcription factor 6 (SOX6) enhances erythroid differentiation in human erythroid progenitors by inducing the expression of B-cell lymphoma-extra larger (Bcl-xL), which has been known to increase in late erythroid differentiation [56]. Another study proposed the involvement of BMP and Wnt signaling pathways in erythropoiesis through their responsive transcription factors—SMAD family member 1 (SMAD1) and transcription factor 7 like 2 (TCF7L2), respectively [57]. In this study, Trompouki et al. demonstrated that the co-occupancy of the cis elements of genes by SMAD1 and TCF7L2, together with a master lineage regulator, such as GATA2 for HSPCs, CCAAT enhancer binding protein alpha (CEBPA) for myeloid lineage and GATA1 for erythroid lineage, was directed by the binding of predominant lineage regulator. GATA1 induction directed the loss of SMAD1/TCF7L2 occupancy on nonerythroid targets and shifted it to enhancers near key erythroid genes in erythroblasts. Translational regulation has been implicated in erythropoiesis. As previously described, DBA is a human anemic disease resulting from the impaired production of full-length GATA1 by pathogenic mutations [47]. Interestingly, in about 50% of DBA patients, the mutations in genes encoding ribosomal proteins (RPs), such as RPL5 and 11, have been found, indicating that altered protein translation, especially GATA1 translation, may be involved in the reduction and defective maturation of erythroid progenitor cells [58]. The mutations in RPS19 caused the decline of GATA1 activity in DBA patients without any change in the mRNA level, indicating that the GATA1 translation has been reduced [59]. Another example can be found in cellular iron metabolism during the later stages of erythropoiesis. When erythroblasts experience iron deficiency, iron regulatory proteins (IRPs) regulate heme biosynthesis and iron uptake by binding to iron responsive elements (IREs) with a stem-loop structure of mRNA UTRs [60].

## 4. Human PSC-Derived Erythroid Lineage Cells

### 4.1. Generation of RBCs from CD34^+^ HSCs and/or HSPCs

Research on the generation of human cultured RBCs has been under way since the end of the 1980s [61,62,63]. The introduction of a liquid culture system has allowed us to promote the proliferation and differentiation of stem cells derived from peripheral blood (PB), umbilical cord blood (CB), and BM into erythrocytes. More-specific and -defined methods of differentiation were subsequently developed using CD34^+^ HSPCs [64,65] (reviewed in detail by [66]). For example, Neildez-Nguyen et al. proposed a culture system that utilized CD34^+^ HSPCs from human CB and yielded a 200,000-fold expansion of pure erythroid precursor cells [67]. While they were not further differentiated into mature RBCs in vitro, these precursor cells infused into a mouse model achieved complete maturation into functional enucleated RBCs, suggesting the importance of microenvironmental factors for full maturation into RBCs. Similarly, Giarratana et al. isolated CD34^+^ HSPCs from human BM, CB, and PB, and induced their proliferation [68]. This study showed a much more potent commitment to the erythroid lineage cells, in which approximately 90% of the CD34^+^ HSPCs exhibited RBC-like characteristics in their maturity and functionality. Notably, these differentiated RBCs were able to transport and release oxygen. In contrast to previous studies [67,68], Miharada et al. developed a method that efficiently produced enucleated RBCs from human CB in the absence of feeder cells [69]. It has been thought that the enucleation of erythroblasts depends on EBIs or niches, such as cell-to-cell and cell-to-extracellular matrix adhesion [70,71,72]. However, their findings suggested that the feeder cell layer is not necessary and that the paracrine factors from EBIs are sufficient for the efficient enucleation of erythroblasts. Lastly, Fujimi et al. combined these approaches to generate RBCs from CD34^+^ HSPCs from human CB [73]. They first cultured CD34^+^ HSPCs on telomerase gene-transduced human stromal cells (hTERT stroma). Using an hTERT stroma, which is of human origin, is safer than using a murine stroma, as the use of animal-derived feeder cells precludes clinical applications. A liquid culture system was next selected to eliminate the hTERT stroma, and erythroblasts were then co-cultured with macrophages to complete the enucleation process [71,74,75]. The removal of macrophages clearly showed a higher enucleation rate [73] than in Miharada et al. [69]. While this has proven to be efficient for producing RBCs from CD34^+^ HSPCs of diverse origin in vitro [67,68,69,73], there are some major shortcomings. This method depends on donations and dedicated blood banks [76]. Since donation-based collection has a limited number of cell sources, it is imperative to find a permanent source of cells to generate RBCs. There are also risks with any donated blood product. A donation-based collection is a system of production in batches, resulting in the problem of batch-to-batch variability and quality of CD34^+^ HSPCs. The last concern is the incompletion of β-globin switching using this method.

### 4.2. Generation of RBCs from hPSCs

The advent of hPSCs has provided a unique opportunity to overcome these shortcomings [5,6]. This unlimited cell source that includes ESCs and iPSCs is capable of self-renewal and differentiation into various cell types. Particularly, many have attempted to obtain hPSC-derived RBCs to lessen the dependence on blood donation (Table 1).

Strategies applied to generate RBCs from CD34^+^ HSPCs from PB, BM and CB were also extended to the differentiation of hPSCs, recapitulating the defining events of erythropoiesis [7]. Different combinations of the key factors, including SCF, EPO, thrombopoietin (TPO), interleukin 3 and 6 (IL3 and IL6), and fms-related receptor tyrosine kinase 3 (FLT3) ligand, were frequently used and optimized to differentiate into RBCs [99,100,101,102,103]. Moreover, many developed a stepwise protocol that first differentiates hPSCs into HSCs via the use of feeder cells, formation of an embryoid body (EB), or monolayer culture system. For example, Olivier et al. obtained and expanded CD34^+^ cells from a population of hESCs [77]. A population of CD34^+^ cells was then cultured in serum-free medium containing insulin, transferrin, IL3, FLT3 ligand, hemin, BMP4, and IGF1, which were then co-cultured with MS5 feeder cells of murine origin, displaying similar results to those of early erythroblasts in 24 days. These hESC-derived erythroblasts were hemoglobinized and expressed a mixture of embryonic and fetal globins, but not adult globins. Dias et al. used the same feeder cells but simpler combinations of SCF, IL3, IL6, EPO, TPO, and colony-stimulating factor 3 (CSF3) to differentiate hESCs and hiPSCs into RBCs [80]. Ma et al. developed two combinations of the factors in which dexamethasone (Dex), SCF, EPO, TPO, IL3, and IL6 were all used, but insulin was added to the first and transferrin and FLT3 ligand were added to the second combination [79]. They then co-cultured hESCs with murine fetal liver-derived stromal cells to generate functional RBCs, which were not only functional oxygen carriers but also expressed GYPA (CD235a) and adult globins, implying a more advanced protocol to mature RBCs by feeders. The Tisdale group developed a novel protocol that first differentiated hPSCs into human yolk sac-like sacs and into RBCs in medium containing SCF, EPO, TPO, IL3, FLT3 ligand, BMP4, and vascular endothelial growth factor (VEGF) [81,82]. Similarly, Cerdan, Rouleau, and Bhatia also demonstrated that the addition of VEGF to a combination of SCF, EPO, IL3, IL6, FLT3 ligand, CSF3, and BMP4 selectively promotes the differentiation of hESCs into early erythroblasts through EB formation [83]. These early erythroblasts expressed embryonic globins. Through EB formation, Chang et al. generated hESC-derived RBCs that expressed both embryonic and fetal globins in the presence of Dex, SCF, EPO, TPO, IL3, IL6, FLT3 ligand, CSF3, transferrin, VEGF, and fibroblast growth factor 2 (FGF2) [84]. A four-step protocol established by Lu et al. aimed to generate RBCs from hESCs [85]. This paper is the first paper comparing the oxygen equilibrium curves of the PSC-derived RBCs with adult RBCs, demonstrating the normal physiologic function of hPSC-derived RBCs. They also introduced a specific cytokine cocktail for erythroid lineage differentiation. The first two steps were the formation and expansion of hematopoietic cells in serum-free medium containing BMP4, VEGF, FGF2, SCF, TPO, FLT3 ligand, and triple protein transduction domain-homeobox B4 (tPTD-HoxB4) fusion protein. The third and last steps were the differentiation and enrichment of RBCs in medium containing only SCF and EPO. The resulting RBCs were enucleated, expressed adult globins, and possessed oxygen-transporting capacity after 28 days of culture. Lapillonne et al. developed a simpler protocol that comprised two steps [86]. In the first step, hPSC-derived EBs were cultured in liquid culture medium containing human plasma, SCF, TPO, FLT3 ligand, BMP4, VEGF, IL3, IL6, and EPO for 20 days. In the second step, the dissociated EBs were treated with human plasma, insulin, heparin, SCF, IL3, and EPO for another 26 days. These RBCs were enucleated and expressed fetal globins in a functional tetrameric globin form, consisting of 43% α-form, 29% γ-form and 5% β-form, suggesting their immature status. Dorn et al. and Olivier et al. developed a protocol for the large-scale production of RBCs through EB formation [87,88]. While different combinations were utilized, the differentiated RBCs were enucleated and expressed embryonic and fetal globins. Interestingly, Olivier et al. used isobutyl methyl xanthine (IBMX), a non-selective inhibitor of cyclic adenosine monophosphate (cAMP) phosphodiesterase, to improve efficiency, as the role of cAMP in HSC regulation and erythropoiesis has previously been reported [104,105]. The Dorn group established a protocol for the generation of CD34^+^ hematopoietic cells, which were further differentiated into GYPA^+^ enucleated RBCs. SCF, IL3, ad EPO were mainly used in this study, which turned out to be a representative cytokine combination for inducing erythropoiesis. Recently, the Kim group differentiated hPSCs into the erythroid lineage cells through EB formation [8,91]. Interestingly, their combinations varied in their two studies. Their first study started with a combination of BMP4, VEGF, wingless-related integration site 3A (WNT3A), activin A, and glycogen synthase kinase 3β inhibitor VIII (GSK3β inhibitor VIII) to form EBs. They then added FGF1, SCF, and β-estradiol for a day and utilized a combination of BMP4, VEGF, FGF1, SCF, IGF2, TPO, heparin, β-estradiol, and IBMX for 11 days to commit EBs to the HSC lineage. They then reduced to a combination of hydrocortisone, SCF, IL3, and EPO, and eventually to poloxamer 188 [8]. In their subsequent study [91], the Kim group started with a combination of BMP4, FGF2, activin A and WNT3A for EB formation, and added VEGF. They then used a combination of hydrocortisone, SCF, IL3, EPO, poloxamer 188, heparin, and human plasma to differentiate into RBCs. While EB-mediated methods of differentiation might provide a microenvironment or niche for the enucleation of erythroblasts, the disadvantages of these methods are the culture-to-culture variability and the large degree of labor intensity [106].

Thus, to avoid this complexity and heterogeneity, monolayer culture systems without EB formation were developed. Particularly, a feeder-free system is indispensable for clinical application. Smith et al. used a two-dimensional (2D) feeder-cell-free culture system optimized to produce RBCs [92]. They developed different combinations of the factors to produce a mixed population of cells that express markers of the megakaryocyte and erythroid lineages. The first combinations they used were BMP4, VEGF, and WNT3A, and then they replaced WNT3A with FGF2 for four to five days. They then used a combination of VEGF, FGF2, SCF, and FLT3 ligands, and then added IL6, TPO, EPO, and 6-formylindolo[3,2-b]-carbazole (FICZ). In addition, they demonstrated that the aryl hydrocarbon receptor (AhR) agonist, FICZ, not only promotes the expansion of HPCs but also drives the specification of both the megakaryocyte and erythroid lineages [92]. Studies showed the AhR plays a critical role in HSC growth and differentiation. To reduce the cost of methods of differentiation, Olivier et al. developed new albumin-free, low-transferrin, and chemically defined culture media [93]. Their novel protocol yielded high rates of enucleation in hiPSC-derived RBCs. The first combination was BMP4, VEGF, WNT3A, WNT 5A, activin A, GSK3β inhibitor VIII, and FGF2, which was followed by the addition of SCF and β-estradiol for three days. The third combination was BMP4, VEGF, FGF2, SCF, TPO, IGF2, β-estradiol, SB431542, IBMX, heparin, and UM171 for hematopoietic specification. After 10 days of culture, the fourth combination of SCF, EPO, IBMX, and Dex was applied for erythroid specification. At day 17, hiPSC-derived erythroblasts were expanded and matured into enucleated RBCs in albumin-free medium containing SCF, EPO, and mifepristone (RU486). This protocol yielded a population of enucleated RBCs that expressed both embryonic and fetal globins. Interestingly, a protocol established by Netsrithong et al. allowed for the differentiation of hiPSCs into a population of multipotent hematoendothelial progenitors (HEPs) that can be further differentiated into endothelial cells (ECs), RBCs, and T lymphocytes [95]. The differentiation of hiPSCs into HEPs was induced by using ascorbic acid, FGF2, VEGF, and CHIR990921 for five days, and hiPSC-HEPs were further induced toward HSPCs in SB431542-added medium up to day 8 or 12. At day 8, hiPSC-derived HSPCs were then differentiated into RBCs in a liquid culture system containing transferrin, SCF, EPO, and IL3 for 20 days. The resulting RBCs expressed GYPA and predominantly produced embryonic and fetal globins with a small amount of adult globin. Since previously established protocols rely on the use of many factors and small molecules [86,96,97], Tursky et al. reported an optimized feeder-cell- and serum-free monolayer culture system by directly comparing with previously established protocols [98]. This 2D-multistep protocol first used the protocol established by Smith et al. to differentiate hiPSCs into CD34^+^ HSPCs [92]. Both Smith et al. and Tursky et al. treated hiPSCs with FICZ to improve their differentiation efficacy. These hiPSC-derived HSPCs were then differentiated into RBCs using a combination of FICZ, BMP4, VEGF, FGF2, SCF, FLT3 ligand, EPO, TPO, and IL6. While these factors were commonly used, they added monothioglycerol, ascorbic acid, and WNT3A. The resulting RBCs expressed embryonic and fetal globins, which were reflective of yolk sac-derived hematopoiesis [107]. While there have been many approaches for the generation of RBCs from hPSCs, additional optimization is necessary for the efficient production of more mature RBCs suitable for human RBC transfusion.

## 5. Current Challenges and Future Directions Using hiPSC-Derived Erythroid Lineage Cells

### 5.1. Heme Synthesis during Erythropoiesis

Hemoglobin (Hb) is commonly known as the iron-bound protein in RBCs that is important to oxygen transfer in humans. RBCs contain Hb, which is a tetrameric protein composed of two α-globin chains and two β-globin chains (HbA, α2β2) attached to Fe^2+^-protoporphyrin [108,109]. Gower1 (ζ2ε2), Gower2 (α2ε2), and Portland (ζ2γ2) are HbF types present at 4–8 weeks of pregnancy (also referred to as embryonic hemoglobin). At 38 weeks, HbF has α2γ2, which has a strong capacity to bind oxygen compared to HbA [18]. Adult hemoglobin in definitive erythroid cells has three Hb types: HbF (α2γ2, <1%), HbA2 (α2δ2, 1–2%), and HbA (α2β2, >98%) [110]. In fetal development, HbF is the main hemoglobin receiving oxygen from the mother’s bloodstream. However, the switch from HbF to HbA gradually occurs, which is critical for normal physiological function after birth. If normal hemoglobin synthesis fails during the conversion of γ2 to β2, the globin disorder beta-thalassemia anemia occurs. Of note, the development of embryonic-derived hematopoiesis in humans differs from that of mice. Whereas β-globin completion in mice is accomplished in the liver stage within 20 days, humans require a longer period for β-globin completion. Since most erythroid lineages with human β-globin are acquired after several months in the BM, the completion of β-globin from human definitive erythropoiesis may differ, compared to that of mice. Additionally, unlike developmental embryogenesis, some controversy exists regarding the events involved in switching from γ-globin to β-globin when culturing erythroid lineages from hPSCs in vitro. Umeda and Qiu et al. reported that the switching from γ-globin to β-globin can occur in both primitive and definitive erythroid cells such as OP9 stromal cells in a feeder cell co-culture system [111,112], whereas Peschle et al. showed that asynchronous switching occurred in hPSC-derived erythroid lineages and that the switching from γ-globin to β-globin can occur by introducing BCL11A-L, rather than by paracrine effects of OP9 cells [54,113,114,115]. This process is involved in transcriptional regulation. The major transcription factors for globin synthesis are GATA1 and KLF1 [116,117,118]. TAL1 is regarded as an essential transcription factor to induce a full maturation with the expression of β-globin [119]. While α-globin expression is derived from primitive erythrocytes, β-globin expression and switching from γ-globin are derived from definitive erythrocytes [112,120,121]. Heme also stimulates globin gene transcription and may be involved in promoting erythroid differentiation [122].

### 5.2. β-Globin Expression in Erythrocytes

One of the biggest challenges in generating functional hPSC-derived RBCs is the stable expression of β-globin. An understanding of the mechanisms that govern the switching from γ-globin to β-globin in humans can help to develop functional hiPSC-derived RBCs as a therapeutic source. Studies of γ-globin suppression have provided insights into how β-globin activation may lead to full maturation and treatment for anemia by revealing the relationship between transcription factors and small molecules [123,124,125,126]. Embryonic and fetal globin genes are autonomously repressed in adult-stage erythroid cells. In particular, MYB, together with SOX6 and GATA1, regulates KLF1 activation, which leads to the suppression of ε-globin. Activated KLF1 inhibits ε- and γ-globin by activating BCL11A, which is a negative mediator of ε- and γ-globin [54,124,127]. BCL11A is now regarded as a crucial activator of HbF silencing in the switch to β-globin [128,129,130]. In the culture systems, to differentiate hPSCs into the erythroid cells, β-globin expression is rare, unlike postnatal erythroid cells, suggesting the status of hPSC-derived erythroid cells as primitive erythroid cells [114,121,131]. Developmental studies have disclosed what happens in mouse definitive erythropoiesis; however, human definitive erythropoiesis is still unclear due to the difficulty of obtaining human samples. It is known that the definitive characteristic of mature RBCs is an increased β-globin level [115,121,132]. Although many studies have been conducted to overcome the issues of ontogeny developmental and fundamental event switching to β-globin from γ-globin [107,133,134], functional β-globin acquisition under in vitro culture conditions cannot be successful without the application of essential niches, such as BM [87,134]. Moreover, because multiple erythropoietic steps can coexist in the differentiation culture conditions, studying the developmental identity of hPSC-derived erythroid cells with β-globin according to diverse protocols for differentiating RBCs from iPSCs, such as 3D organoid application, is challenging. Early protocols for RBC differentiation mainly reported the cell generation to primitive erythroid by using stromal cells, such as fetal liver cells and OP9 cells, which led to inefficiency in β-globin expression [80,111]. However, secretory factors from OP9 stromal cells are not enough to fully differentiate into the erythroid lineage cells, such as adult RBCs [135]. Recent molecular studies reported technological advances in developing and validating culture conditions to increase the expression of β-globin [115,134,136]. The establishment of hiPSCs with gene editing is emerging as a technically advanced tool to treat globin disorders by the autologous transplantation of gene-edited hiPSC-derived erythroid lineage cells based on success with mouse iPSC gene editing [137].

### 5.3. Iron Supplementation for Erythrocytes

In erythropoiesis, adequate supplies of folate, vitamin B12, and iron are required. Among them, iron is an indispensable element for living cells. Iron homeostasis in mammals is mainly regulated by regulatory systems, including iron IRP/IRE in cytoplasmic iron homeostasis, which is a recently emerging factor; hepcidin ferroportin (SLC40A1; FPN1) in serum levels; and HIF2α transcriptional regulation [138,139,140]. SLC40A1 and HIF2α are well-established factors in iron homeostasis, whereas the involvement of IRPs in cellular and systemic iron homeostasis was shown more recently [141,142,143,144]. IRPs can regulate gene expression involved in iron metabolism by binding to IRE in the target mRNA, increase cellular iron absorption, and decrease the storage and export of iron to maintain iron balance. There are two IRP families: IRP1 and IRP2. Their functional phenotypes were addressed in knockout mice and are regarded as predominant factors in erythropoiesis as well as iron homeostasis. Mice with deficiencies of both IRP1 and IRP2 are not viable, but mice with either IRP1 or IRP2 deficiency are fertile, implying that IRPs can compensate for each other and are functionally redundant [145,146]. Erythroblasts are the most avid consumers of iron. They maintain high transferrin receptors, but a deficiency of IRP2 decreases transferrin receptors, resulting in an insufficient iron cycle. A deficiency of IRP2 leads a reduced expression of the aminolaevulinic acid synthase (ALAS) enzyme, ultimately inducing microcytic hypochromic anemia by protoporphyrin IX accumulation [147]. Although the transferrin amount is high in plasma, iron storage within BM is absent, and erythroid precursors in IRP2 deficiency display a low number of transferrin receptors, resulting in the failure of iron homeostasis. IRP proteins can alter their expression and function according to iron levels. Thus, IRP proteins in erythroblasts should also be confirmed in cultured hPSC-derived RBCs alongside iron level. When culturing hPSC-derived RBCs, iron is regarded as a crucial factor for increasing erythroblasts. Most cells, including erythroblasts, can uptake iron from transferrin. The delivery of iron to erythroblasts mainly occurs by the binding transferrin to transferrin receptors [80,148]. Transferrin exists in three forms; holo-transferrin, which is known as transferrin bearing two ferric irons; apo-transferrin, which maintains non-iron loading status; and partial iron-loaded transferrin, which is one iron-loaded transferrin. Holo-transferrin can bind to both transferrin receptors 1 and 2 (TfR1 and 2) in erythroid precursors, mediate iron uptake via endocytosis with TfR1, and stimulate erythropoietic signaling through TfR2. Two ferric irons (FeIII) can be released from transferrin by the reduction to FeII, facilitated by endosomal ferric reductase in the cytosol [149]. After releasing iron into the cytosol, transferrin is exported on the membrane as apo-transferrin and binds to ferric iron again, leading to holo-transferrin. This in vivo ferric iron and ferrous iron circulation is reversibly governed by numerous proteins. Erythroblast heme levels regulate the uptake of iron and globin biosynthesis, cooperating with numerous proteins, including IRP/IRE, SLC11A2, and STEAP3 metalloreductase (STEAP3), in iron absorption, storage, export, and other functions [141,150,151].

### 5.4. Enucleation of OrthoE

Enucleation is a key feature that characterizes the terminal maturation of RBCs, creating reticulocytes and free nuclei. In definitive erythropoiesis, arising in the fetal liver and in the BM after birth, erythroblasts extrude the nucleus before entering the circulation, whereas in primitive erythropoiesis, such as cells emerging from the yolk sac during embryonic development, nucleated erythroid cells enter the bloodstream and progressively mature [152,153]. During erythropoiesis, cells undergo a series of changes, producing enucleated and functional RBCs [154]. At the end of the maturation process, in OrthoE, cell cycle arrests and nuclear and chromatin condensation occur, followed by nuclear polarization and macrophage interactions [155]. The resulting nuclear expulsion from the OrthoE generates reticulocytes, consisting mostly of the cytoplasm, and pyrenocytes with the condensed nuclei [153]. Reticulocytes then further develop into functional RBCs, while pyrenocytes with phosphatidylserine signals on their plasma membrane are rapidly engulfed by the macrophages of the EBIs [43,156]. Enucleation is a complex process that requires fine regulation for terminal maturation in RBC production. Enucleation is tightly controlled by concomitant mechanisms of cell cycle arrest, chromatin condensation, and nuclear polarization. Cell cycle arrest is a critical step initiating enucleation. KLF1 directly targets E2F transcription factor 2 (E2F2) during terminal erythropoiesis and plays a pivotal role in enucleation by inducing the expression of citron Rho-interacting kinase (CIRK), which controls microtubule organization and cytokinesis [157,158,159]. Chromatin and nuclear condensation is another essential precondition for enucleation and is dependent on histone acetylation levels. General control non-depressible 5 (GCN5), a histone acetyl transferase (HAT), is downregulated, and the acetylation of histone H3 lysine 9 (H3K9ac) and histone H4 lysine 5 (H4K5ac) is decreased during mouse fetal erythropoiesis [160,161]. In addition, histone deacetylase 2 (HDAC2) affects chromatin condensation and pyknosis, enhancing contractile actin ring (CAR) formation during enucleation [162]. Major histones are also released during erythroblast enucleation through a nuclear opening induced by caspase 3 activity-dependent lamin B cleavage and chromatin condensation [158]. Moreover, the ectopic expression of miR-191 or knockdown of its target genes hampered enucleation via the inhibition of chromatin condensation [163]. Additionally, nuclear polarization is a crucial feature that precedes enucleation. It occurs in a similar manner to cytokinesis but in an asymmetric way during enucleation. It was demonstrated that the downregulation of Ras-related C3 botulinum toxin substrate 1 (Rac1) GTPase and mDia2 Rho GTPase disrupts CAR formation and blocks erythroblast enucleation [164]. Moreover, clathrin-mediated vesicle formation, near the nuclear extrusion site in both murine and human erythroblasts, is required for a proper enucleation [165]. Survivin also contributes to enucleation through the interaction of epidermal growth factor receptor substrate 15 (EPS15) and clathrin [166]. Various molecular players have been highlighted in recent years and yet most of them are not from human erythropoiesis. Thus, studies of the molecular mechanism underlying the enucleation process in humans are still at their early stage.

### 5.5. Methods for Enucleation of hPSC-Derived OrthoE

Various studies have demonstrated that hPSC-derived erythroblasts are capable of enucleation in vitro. Qiu et al. established an hESC-derived RBC differentiation protocol by co-culturing with immortalized fetal hepatocytes [114]. The average enucleation rate from their method reached about 6.5% by extending the culture duration to 35 days, whereas no enucleated cells were detectable at day 14. Moreover, Lu et al. generated erythroid cells from four hESC lines by EB formation and co-culturing with human mesenchymal stem cells (MSCs) or OP9 mouse stromal cells [85]. They showed chromatin condensation, extrusion of the pyknotic nucleus, and a similar diameter of erythrocytes compared to normal RBCs. They showed that their method is suitable for the large-scale production of RBCs and improves enucleation efficiency up to 65% on OP9 feeder cell layers. On the other hand, they obtained 10~30% enucleated erythroblasts using the Matrigel system, instead of mouse embryonic fibroblasts (MEFs), suggesting that a feeder-free system is also attainable for enucleation. Lapillonne et al. differentiated RBCs from both hiPSC and hESC lines [86]. As a result, 4% to 10% and 52% to 66% of enucleated RBCs were obtained from hiPSC and hESC lines, respectively. Dias et al. also described the erythrocyte differentiation of hiPSCs and hESCs [80]. With their approach, the enucleation rates from hiPSC- and hESC-derived erythroid cells were similar, but both were as low as 2% to 10%. In 2015, Dorn et al. observed that 21% to 29% of enucleated erythrocytes are differentiated from various hiPSC lines [87]. Erythroid cells were differentiated from CB cell-derived, neural stem cell-derived, and fibroblast-derived iPSCs in addition to H1-ESCs. The differential potentials for RBC generation were investigated in association with the epigenetic memory of source cells before hiPSCs reprogramming. All hiPSC lines underwent terminal maturation steps, although the growth rate of erythrocytes varied. More recently, Olivier et al. established a culture system using combinations of growth factors and small molecules that are compatible for clinical application. They manufactured hiPSC- and hESC-derived erythroid cells under serum-free and feeder-free conditions in a good manufacturing practice (GMP) facility. However, their system is complex, and limited by low enucleation rates below 10% [88]. In 2019, an updated protocol from the same group with a higher expansion rate and about a 42% enucleation efficiency was reported [93]. This protocol, called the “long PSC-RED” protocol, describes chemically defined, albumin-free, and less-transferrin culture conditions for erythroid differentiation. Moreover, a filtration step was added to eliminate nucleated cells and the expelled nuclei, which leads to more than 94% of enucleated cells in the final population. As enucleation is a rate-limiting step for RBC maturation, achieving efficient differentiation into enucleated functional RBC populations needs to be more extensively investigated for in-human applications.

### 5.6. Future Directions of Clinical Applications Using hiPSC-Derived Erythrocytes

To transfuse in-vitro-manufactured RBCs in humans, safety and large-scale production are the major concerns. Although pioneer studies using hPSCs for the expansion of mature RBCs struggled with less than 10% enucleation rates on average, studies of RBC generation from HSCs and HSPCs have focused on the large-scale production and purification of enucleated erythroid cells. Giarratana et al. developed a liquid culture system of erythroid cells derived from human CD34^+^ cells, which were co-cultured with MS5 or MSC layers [68]. It was the first report of a large-scale expansion of cultured RBCs with more than a 65% enucleation rate. The authors highlighted that the presence of the microenvironment was essential for the terminal maturation of erythroid cells and hemoglobin synthesis. A year later, Miharada et al. reported that the in vitro culture of CB-derived RBCs without a feeder layer was possible in a large-scale system, and nearly 80% of the generated erythrocytes were enucleated [69]. Furthermore, Timmins et al. obtained over 500 units of RBCs in a stirred bioreactor setting, with more than 90% of enucleation in the absence of SCF and hydrocortisone in the culture [167]. On the other hand, efforts to enhance the enucleation efficiency of hPSC-derived RBCs are still in progress. According to Dorn et al., a percentage of enucleated cells from hiPSC-derived erythrocytes were below 29%, whereas adult CD34^+^CD45^+^ HSCs cultured under the same condition resulted in homogeneous differentiation, with 85.2% enucleated RBCs [87]. Bernecker et al. also established a hematopoietic cell-forming complex (HCFC)-based protocol that gives mean enucleation rates near 40% from both CD34-hiPSCs (CD34^+^ CB) and PEB-iPSC (CD36^+^ BasoE) [168]. To represent biological replicates, the hiPSC lines were generated from CB-derived CD34^+^ cells (CD34-hiPSCs) and CD36^+^ BasoE (PEB-iPSCs) before HCFC formation. To overcome the hurdle, cultured RBCs were purified on day 18 to remove extruded nuclei and nucleated erythroblasts, thus reaching ~98% purity of enucleated cells. They conducted a comprehensive functional characterization of enucleated hiPSC-derived RBCs in comparison with native reticulocytes and native RBCs for morphology, biomechanical characteristics, membrane composition, and blood group antigen expression. According to Olivier et al., the purification of hiPSC-derived cultured RBCs yielded a ~99% population of enucleated cells [93]. They suggested that an enucleation rate greater than 40% is sufficient to produce pure populations of enucleated RBCs, to achieve an industrial scale. Altogether, feeder-free, xenogeneic material-free, and large-scale culturing with highly purified enucleated RBCs is now available for clinical applications, while some concerns remain.

## 6. Conclusions

To date, there is no substitute to blood transfusion, despite the substantial efforts made in the generation of blood using various stem cells. Among hPSCs, hiPSC can be a more universal source for RBC generation, as hESCs raise ethical and political questions. For RBC generation, a proper understanding of primitive and definitive erythropoiesis during development is necessary. This review covers diverse aspects of erythroid lineage differentiation for regenerative medicine and describes the latest technical advancements in generating functional RBCs. In an attempt to elucidate the fine-tuning machinery of erythrocyte maturation, studies on cellular and molecular mechanisms beyond erythropoiesis have grown over the last decade. Particularly, the microenvironmental niches, such as EBIs, play a crucial role for generating RBCs that exhibit an adult phenotype. More importantly, various culture systems for the in vitro generation of functional RBCs from hPSCs have been developed: the use of feeder cells, formation of EB, or a monolayer culture system using a xeno-free system. While hPSCs are a valuable source for the artificial production of RBCs, other challenges remain for clinical application of hPSC-derived RBCs, including efficiency, sustainability, viability, and functionality. For example, differentiation efficiency varies between the cell lines due to the use of donor specificity and epigenetic influences. Therefore, an autologous approach may be more difficult than an allogeneic one. The biggest challenge for clinical application is the efficiency of the differentiation protocols. In general, most protocols are still short of generating a sufficient number of functional RBCs. Low efficiency in RBC differentiation is mainly due to the insufficient terminal maturation of erythroblasts, including stable β-globin expression and enucleation. It is not only the number of the produced RBCs that matters, but also the function and homogeneity. Thus, developing more efficient protocols that can also generate functional and homogeneous RBC populations is necessary. For clinical application, the methods for mass production, to obtain a sufficient yield of functional RBCs, are mandated. The usage of advanced engineering technologies, such as bioreactors, may lead to the possibility of large-scale manufacturing. At the same time, cost-effectiveness needs to be accounted for. The use of multiple cytokines and growth factors for over 40 days on average, including HSC differentiation steps (summarized in Table 1), is challenging for industrial application. For clinical use, the materials should be in GMP grades, which are much more expensive than research-grade counterparts. Essentially, setting up reliable and recognized quality control systems and criteria compatible for in-human use is required. Methods for the molecular characterization and functional assessment, to test the identity, purity, and potency of the generated cells, must be standardized, in addition to the sterility and stability controls during and after manufacturing. Notwithstanding, there has been enormous progress in the generation of hPSC-derived RBCs, and thus, hiPSC-RBCs are at the final stages before clinical trials.

## Figures and Tables

**Table 1:** Summary on differentiation of hPSCs into erythrocytes.

Culture System		Source Cell Type	Differentiated Cell Type(s)	Factor(s)	Culture Duration	Molecular Characterization	Functional Assessment	Note	Reference
Feeder cell	MS5	hESC	Early erythroblast	Insulin, transferrin, IL3, BMP4, FLT3 ligand, SCF, EPO, IGF1, hemin	24 days (+15 days for HSC differentiation)	CD34^+^GYPA^+^TFRC^+^, orthochromatic, embryonic and fetal globin	Hemoglobin production	Large-scale production of RBCs	[77]
	mFLSC	hESC	RBC	SCF, IL3, IL6, EPO, TPO, CSF3	18 days	GYPA^+^TFRC^+^, clonogenic, embryonic, fetal, and adult globin	Oxygen dissociation curve	Adapted from [78]	[79]
	MS5	hiPSC, hESC	RBC	Dex, insulin, SCF, EPO, TPO, IL3, IL6	40–45 days (+7–8 days for HSC differentiation)	CD34^+^SPN^+^GYPA^+^TFRC^+^, embryonic and fetal globin	N/A		[80]
	OP9	hiPSC, hESC	RBC	SCF, FLT3 ligand, EPO, TPO, IL3, BMP4; VEGF	15 days (+15 days for sac differentiation)	CD34^+^ PTPRC^+^GYPA^+^, clonogenic, embryonic, fetal, and adult globin	N/A	Gene correction in patient-iPSC from [81]	[82]
	VEGF, BMP4, SCF, FLT3 ligand, IL3, IL6, CSF3, EPO	hESC	Erythroid precursor, early erythroblast	VEGF, BMP4, SCF, FLT3 ligand, IL3, IL6, CSF3	15 days (+15 days for EB formation)	CD34^+^PTPRC^+^KDR^+^GYPA^+^, embryonic globin, clonogenic, self-renewal	N/A	Importance of VEGF	[83]
EB	Transferrin, ascorbic acid, FGF2, VEGF	hESC	RBC	Dex, EPO, TPO, SCF, FLT3 ligand, IL3, IL6, CSF3, transferrin, FGF2, VEGF	13–29 days (+14 days for EB formation)	CD34^+^GYPA^+^, clonogenic, embryonic and fetal globin	N/A		[84]
	BMP4, VEGF, FGF2, SCF, TPO, FLT3 ligand; FGF2, tPTD-HoxB4	hESC	RBC	EPO, SCF	21 days (+3–5 days for EB formation; +10 days for blast colony formation and expansion)	TFRC^+^GYPA^+^CD47^+^, embryonic and fetal globin	Oxygen dissociation curve	Blast colony formation; enucleation on days 36–42	[85]
	SCF, TPO, FLT3 ligand, BMP4, VEGF, IL3, IL6, EPO	hiPSC, hESC	RBC	Insulin, heparin, SCF, IL3, EPO, human plasma	25 days (+20 days for EB formation)	CD34^+^PTPRC^+^TFRC^+^GYPA^+^, enucleated/orthochromatic, fetal globin	Hemoglobin production, hemoglobin allosteric transition	Adapted from [67,68]	[86]
	SCF, TPO, FLT3 ligand, IL3, IL6, VEGF, BMP4, EPO	hiPSC, hESC	RBC	Human plasma, insulin, transferrin, SCF, IL3	25 days (+20 days for EB formation)	Enucleated, embryonic and fetal globin	N/A		[87]
	BMP4, VEGF, activin A, WNT3A, GSK3βi VIII, FGF1, SCF, β-estradiol	hiPSC, hESC	RBC	BMP4, VEGF, FGF1, IGF2, TPO, heparin, IBMX, β-estradiol, hydrocortisone, FLT3 ligand, IL3, IL11, IGF1	24–31 days (+2 days for EB formation)	CD36^+^GYPA^+^, enucleated, embryonic and fetal globin	N/A	cGMP-compatible	[88]
	BMP4, VEGF, FLT3 ligand, IL3, IL6, SCF, TOP, EPO	hiPSC	RBC	IL3, SCF, EPO	18 days (+21 days for EB formation)	CD36^+^SPN^+^GYPA^+^, clonogenic, enucleated, fetal and adult globin	N/A		[89,90]
	ROCKi, BMP4, VEGF, WNT3A, FGF1, SCF, activin A, GSK3βi VIII, β-estradiol; FGF2	hiPSC, hESC	RBC	BMP4, SCF, VEGF, IGF2, FGF1, TPO, heparin, EPO, IBMX, β-estradiol, hydrocortisone, IL3, ferric nitrate, poloxamer 188; FGF2, human plasma	24 days (+3 days for EB formation)	SPN^+^TFRC^+^GYPA^+^, enucleated/orthochromatic, fetal globin	Oxygen dissociation curve	Further modified by [8]	[91]
Monolayer	Matrigel-coated, FGF2, ROCKi	hiPSC	RBC	Human plasma-mimetic, FICZ, EPO, BMP4, VEGF, WNT3A, FGF2, SCF, FLT3 ligand, TPO, IL6, ascorbic acid	60 days (+10–15 days for HSC differentiation)	GYPA^+^, embryonic and fetal globin	Hypoxia	Importance of aryl hydrocarbon receptor	[92]
	Vitronectin-coated, FGF2, BMP4, SCF, VEGF, WNT3A, WNT5A, activin A, GSK3βi VIII, β-estradiol	hiPSC	RBC	BMP4, SCF, FGF2, TPO, VEGF, IGF2, β-estradiol, SB431542, heparin, IBMX, EPO, UM171, Dex, RU486, Optiferrin	32–46 days (+7 days for HSC differentiation)	CD36^+^SPN^+^ TFRC^+^GYPA^+^, enucleated/orthochromatic, embryonic, fetal, and adult globin	N/A	Adapted from [88]	[93]
	Matrigel-coated, ROCKi	hiPSC	RBC	Ascorbic acid, FGF2, CHIR990921, VEGF, SCF, SB431542, transferrin, IL3, EPO	20 days (+8–12 days of HSPC differentiation)	CD34^+^GYPA^+^SLC4A1^+^, clonogenic, embryonic, fetal, and adult globin	N/A	Adapted from [94]	[95]
	ECM-coated	hiPSC	RBC	Monothioglycerol, SCF, ascorbic acid, FGF2, WNT3A, IL3, BMP4, EPO, VEGF, TPO, FLT3 ligand, FICZ, IGF1, Dex	16–17 days (+16 days for HSC differentiation)	CD34^+^PTPRC^+^GYPA^+^, clonogenic, embryonic and fetal globin	N/A	Direct comparison of four methods from [86,92,96,97]	[98]

Abbreviations—BMP4 = bone morphogenetic protein 4; CSF3 = colony stimulating factor 3; Dex = dexamethasone; EB = embryoid body; ECM = extracellular matrix; EPO = erythropoietin; FGF = fibroblast growth factor; FICZ = 6-formylindolo[3,2-b]-carbazole; FLT3 = fms-related receptor tyrosine kinase 3; GSK3βi VIII = glycogen synthase kinase 3β inhibitor VIII; GYPA (CD235A) = glycophorin A; hESC = human embryonic stem cell; hiPSC = human induced pluripotent stem cell; HSC = hematopoietic stem cell; HSPC = hematopoietic stem/progenitor cell; IBMX = isobutyl methyl xanthine; IGF = insulin like growth factor; IL = interleukin; KDR = kinase insert domain receptor; mFLSC = mouse fetal liver-derived stromal cell line; MS5 = mouse bone marrow stromal cell line; N/A = not applicable; OP9 = mouse bone marrow stromal cell line; PTPRC (CD45) = protein tyrosine phosphatase receptor type 3; RBC = red blood cell; ROCKi = Rho kinase inhibitor; SCF = stem cell factor; SLC4A1 (AE1, BND3) = solute carrier family 4 member 1; SPN (CD43) = sialophorin; TFRC (CD71) = transferrin receptor; TPO = thrombopoietin; tPTD-HoxB4 = triple protein transduction domain-homeobox B4; VEGF = vascular endothelial growth factor; WNT = Wingless-related integration site.

## Data Availability

Not applicable.

## Abbreviations

Official NCBI gene symbols are used in this review for the sake of consistency.

Abbreviation	Full name
ACVR1B	Activin A receptor type 1B
BCL-xL	B-cell lymphoma-extra large
BCL11A	BCL11 transcription factor A
BMP4, 11	Bone morphogenetic protein 4 and 11
CEBPA	CCAAT enhancer binding protein alpha
EPO, EPOR	Erythropoietin, erythropoietin receptor
ERK	Extracellular regulated MAP kinase
FOXO3	Forkhead box O3
GATA1	GATA binding protein 1
GYPA (CD235A; GPA)	Glycophorin A
IGF1, 2	Insulin like growth factor 1 and 2
IRE	Iron responsive elements
IRF2, 6	Interferon regulatory factor 2 and 6
JAK2	Janus kinase 2
KIT	KIT proto-oncogene, receptor tyrosine kinase
KLF1	LIM domain binding 1
LDB1	LIM domain binding 1
LMO1, 2	Krüppel-Like Factor 1
MEK	MAP kinase-ERK kinase
MYB	MYB proto-oncogene, transcription factor
PI3K	Phosphatidylinositol 3-kinase
RAS	Rat sarcoma
SCF	Stem cell factor
SCL	Stem cell leukemia protein
SLC4A1 (BND3), 11A2 (DMT1), 40A1 (FPN1)	Solute carrier family 4 member 1, 11 member 2, and 40 member 1
SMAD1	SMAD family member 1
SOX6	SRY-box transcription factor 6
SPI1 (PU.1)	Spi-1 proto-oncogene
SPN (CD43)	Sialophorin
STAT5	Signal transducer and activator of transcription 5
TAL1	TAL bHLH transcription factor 1, erythroid differentiation factor
TCF7L2	Transcription factor 7 like 2
TFRC (CD71)	Transferrin receptor
TGFβ	Transforming growth factor B
TNFα	Tumor necrosis factor A
VEGF	Vascular endothelial growth factor
WNT3A	Wnt family member 3A
ZFPM1 (FOG1)	Zinc finger protein, FOG family member 1

## References

[1] Greinacher A., Fendrich K., Hoffmann W. (2010). Demographic Changes: The Impact for Safe Blood Supply. Transfus. Med. Hemother..

[2] Douay L., Andreu G. (2007). Ex vivo production of human red blood cells from hematopoietic stem cells: What is the future in transfusion?. Transfus. Med. Rev..

[3] Migliaccio A.R., Whitsett C., Migliaccio G. (2009). Erythroid cells in vitro: From developmental biology to blood transfusion products. Curr. Opin. Hematol..

[4] Thomson J.A., Itskovitz-Eldor J., Shapiro S.S., Waknitz M.A., Swiergiel J.J., Marshall V.S., Jones J.M. (1998). Embryonic stem cell lines derived from human blastocysts. Science.

[5] Takahashi K., Tanabe K., Ohnuki M., Narita M., Ichisaka T., Tomoda K., Yamanaka S. (2007). Induction of pluripotent stem cells from adult human fibroblasts by defined factors. Cell.

[6] Yu J., Vodyanik M.A., Smuga-Otto K., Antosiewicz-Bourget J., Frane J.L., Tian S., Nie J., Jonsdottir G.A., Ruotti V., Stewart R. (2007). Induced pluripotent stem cell lines derived from human somatic cells. Science.

[7] Dzierzak E., Philipsen S. (2013). Erythropoiesis: Development and differentiation. Cold Spring Harb. Perspect. Med..

[8] Park Y.J., Jeon S.H., Kim H.K., Suh E.J., Choi S.J., Kim S., Kim H.O. (2020). Human induced pluripotent stem cell line banking for the production of rare blood type erythrocytes. J. Transl. Med..

[9] Lim Z.R., Vassilev S., Leong Y.W., Hang J.W., Renia L., Malleret B., Oh S.K. (2021). Industrially Compatible Transfusable iPSC-Derived RBCs: Progress, Challenges and Prospective Solutions. Int. J. Mol. Sci..

[10] Caulier A.L., Sankaran V.G. (2022). Molecular and cellular mechanisms that regulate human erythropoiesis. Blood.

[11] Palis J. (2014). Primitive and definitive erythropoiesis in mammals. Front. Physiol..

[12] Barminko J., Reinholt B., Baron M.H. (2016). Development and differentiation of the erythroid lineage in mammals. Dev. Comp. Immunol..

[13] Yang L., Lewis K. (2020). Erythroid Lineage Cells in the Liver: Novel Immune Regulators and Beyond. J. Clin. Transl. Hepatol..

[14] Chen K., Liu J., Heck S., Chasis J.A., An X., Mohandas N. (2009). Resolving the distinct stages in erythroid differentiation based on dynamic changes in membrane protein expression during erythropoiesis. Proc. Natl. Acad. Sci. USA.

[15] Palis J., Yoder M.C. (2001). Yolk-sac hematopoiesis: The first blood cells of mouse and man. Exp. Hematol..

[16] Palis J., Robertson S., Kennedy M., Wall C., Keller G. (1999). Development of erythroid and myeloid progenitors in the yolk sac and embryo proper of the mouse. Development.

[17] Kingsley P.D., Malik J., Fantauzzo K.A., Palis J. (2004). Yolk sac-derived primitive erythroblasts enucleate during mammalian embryogenesis. Blood.

[18] Kingsley P.D., Malik J., Emerson R.L., Bushnell T.P., McGrath K.E., Bloedorn L.A., Bulger M., Palis J. (2006). “Maturational” globin switching in primary primitive erythroid cells. Blood.

[19] van Deurs B., Behnke O. (1973). The microtubule marginal band of mammalian red blood cells. Z. Anat. Entwickl..

[20] Sangiorgi F., Woods C.M., Lazarides E. (1990). Vimentin downregulation is an inherent feature of murine erythropoiesis and occurs independently of lineage. Development.

[21] Ema H., Nakauchi H. (2000). Expansion of hematopoietic stem cells in the developing liver of a mouse embryo. Blood.

[22] Kumaravelu P., Hook L., Morrison A.M., Ure J., Zhao S., Zuyev S., Ansell J., Medvinsky A. (2002). Quantitative developmental anatomy of definitive haematopoietic stem cells/long-term repopulating units (HSC/RUs): Role of the aorta-gonad-mesonephros (AGM) region and the yolk sac in colonisation of the mouse embryonic liver. Development.

[23] Mikkola H.K., Orkin S.H. (2006). The journey of developing hematopoietic stem cells. Development.

[24] Xue L., Cai J.Y., Ma J., Huang Z., Guo M.X., Fu L.Z., Shi Y.B., Li W.X. (2013). Global expression profiling reveals genetic programs underlying the developmental divergence between mouse and human embryogenesis. BMC Genom..

[25] Popescu D.M., Botting R.A., Stephenson E., Green K., Webb S., Jardine L., Calderbank E.F., Polanski K., Goh I., Efremova M. (2019). Decoding human fetal liver haematopoiesis. Nature.

[26] Shemin D., Rittenberg D. (1946). The life span of the human red blood cell. J. Biol. Chem..

[27] Kuhrt D., Wojchowski D.M. (2015). Emerging EPO and EPO receptor regulators and signal transducers. Blood.

[28] Wu H., Liu X., Jaenisch R., Lodish H.F. (1995). Generation of committed erythroid BFU-E and CFU-E progenitors does not require erythropoietin or the erythropoietin receptor. Cell.

[29] Singbrant S., Russell M.R., Jovic T., Liddicoat B., Izon D.J., Purton L.E., Sims N.A., Martin T.J., Sankaran V.G., Walkley C.R. (2011). Erythropoietin couples erythropoiesis, B-lymphopoiesis, and bone homeostasis within the bone marrow microenvironment. Blood.

[30] Malik J., Kim A.R., Tyre K.A., Cherukuri A.R., Palis J. (2013). Erythropoietin critically regulates the terminal maturation of murine and human primitive erythroblasts. Haematologica.

[31] Nocka K., Majumder S., Chabot B., Ray P., Cervone M., Bernstein A., Besmer P. (1989). Expression of c-kit gene products in known cellular targets of W mutations in normal and W mutant mice—Evidence for an impaired c-kit kinase in mutant mice. Genes Dev..

[32] Wu H., Klingmuller U., Besmer P., Lodish H.F. (1995). Interaction of the erythropoietin and stem-cell-factor receptors. Nature.

[33] Papayannopoulou T., Brice M., Blau C.A. (1993). Kit ligand in synergy with interleukin-3 amplifies the erythropoietin-independent, globin-synthesizing progeny of normal human burst-forming units-erythroid in suspension cultures: Physiologic implications. Blood.

[34] Wang J., Tang Z.Y., Ka W., Sun D., Yao W., Wen Z., Chien S. (2007). Synergistic effect of cytokines EPO, IL-3 and SCF on the proliferation, differentiation and apoptosis of erythroid progenitor cells. Clin. Hemorheol. Microcirc..

[35] Dussiot M., Maciel T.T., Fricot A., Chartier C., Negre O., Veiga J., Grapton D., Paubelle E., Payen E., Beuzard Y. (2014). An activin receptor IIA ligand trap corrects ineffective erythropoiesis in beta-thalassemia. Nat. Med..

[36] Suragani R.N., Cadena S.M., Cawley S.M., Sako D., Mitchell D., Li R., Davies M.V., Alexander M.J., Devine M., Loveday K.S. (2014). Transforming growth factor-beta superfamily ligand trap ACE-536 corrects anemia by promoting late-stage erythropoiesis. Nat. Med..

[37] Mohandas N., Prenant M. (1978). Three-dimensional model of bone marrow. Blood.

[38] Sonoda Y., Sasaki K. (2012). Hepatic extramedullary hematopoiesis and macrophages in the adult mouse: Histometrical and immunohistochemical studies. Cells Tissues Organs.

[39] May A., Forrester L.M. (2020). The erythroblastic island niche: Modeling in health, stress, and disease. Exp. Hematol..

[40] Paulson R.F., Hariharan S., Little J.A. (2020). Stress erythropoiesis: Definitions and models for its study. Exp. Hematol..

[41] Bessis M. (1958). [Erythroblastic island, functional unity of bone marrow]. Rev. Hematol..

[42] Rhodes M.M., Kopsombut P., Bondurant M.C., Price J.O., Koury M.J. (2008). Adherence to macrophages in erythroblastic islands enhances erythroblast proliferation and increases erythrocyte production by a different mechanism than erythropoietin. Blood.

[43] Yoshida H., Kawane K., Koike M., Mori Y., Uchiyama Y., Nagata S. (2005). Phosphatidylserine-dependent engulfment by macrophages of nuclei from erythroid precursor cells. Nature.

[44] Yeo J.H., Lam Y.W., Fraser S.T. (2019). Cellular dynamics of mammalian red blood cell production in the erythroblastic island niche. Biophys. Rev..

[45] Porcher C., Chagraoui H., Kristiansen M.S. (2017). SCL/TAL1: A multifaceted regulator from blood development to disease. Blood.

[46] Xu J., Shao Z., Glass K., Bauer D.E., Pinello L., Van Handel B., Hou S., Stamatoyannopoulos J.A., Mikkola H.K., Yuan G.C. (2012). Combinatorial assembly of developmental stage-specific enhancers controls gene expression programs during human erythropoiesis. Dev. Cell..

[47] Sankaran V.G., Ghazvinian R., Do R., Thiru P., Vergilio J.A., Beggs A.H., Sieff C.A., Orkin S.H., Nathan D.G., Lander E.S. (2012). Exome sequencing identifies GATA1 mutations resulting in Diamond-Blackfan anemia. J. Clin. Investig..

[48] Borg J., Patrinos G.P., Felice A.E., Philipsen S. (2011). Erythroid phenotypes associated with KLF1 mutations. Haematologica.

[49] Drissen R., von Lindern M., Kolbus A., Driegen S., Steinlein P., Beug H., Grosveld F., Philipsen S. (2005). The erythroid phenotype of EKLF-null mice: Defects in hemoglobin metabolism and membrane stability. Mol. Cell. Biol..

[50] Kitajima K., Zheng J., Yen H., Sugiyama D., Nakano T. (2006). Multipotential differentiation ability of GATA-1-null erythroid-committed cells. Genes Dev..

[51] Hodge D., Coghill E., Keys J., Maguire T., Hartmann B., McDowall A., Weiss M., Grimmond S., Perkins A. (2006). A global role for EKLF in definitive and primitive erythropoiesis. Blood.

[52] Gutierrez L., Tsukamoto S., Suzuki M., Yamamoto-Mukai H., Yamamoto M., Philipsen S., Ohneda K. (2008). Ablation of Gata1 in adult mice results in aplastic crisis, revealing its essential role in steady-state and stress erythropoiesis. Blood.

[53] Wontakal S.N., Guo X., Smith C., MacCarthy T., Bresnick E.H., Bergman A., Snyder M.P., Weissman S.M., Zheng D., Skoultchi A.I. (2012). A core erythroid transcriptional network is repressed by a master regulator of myelo-lymphoid differentiation. Proc. Natl. Acad. Sci. USA.

[54] Sankaran V.G., Menne T.F., Xu J., Akie T.E., Lettre G., Van Handel B., Mikkola H.K., Hirschhorn J.N., Cantor A.B., Orkin S.H. (2008). Human fetal hemoglobin expression is regulated by the developmental stage-specific repressor BCL11A. Science.

[55] Stadhouders R., Aktuna S., Thongjuea S., Aghajanirefah A., Pourfarzad F., van Ijcken W., Lenhard B., Rooks H., Best S., Menzel S. (2014). HBS1L-MYB intergenic variants modulate fetal hemoglobin via long-range MYB enhancers. J. Clin. Investig..

[56] Cantu C., Ierardi R., Alborelli I., Fugazza C., Cassinelli L., Piconese S., Bose F., Ottolenghi S., Ferrari G., Ronchi A. (2011). Sox6 enhances erythroid differentiation in human erythroid progenitors. Blood.

[57] Trompouki E., Bowman T.V., Lawton L.N., Fan Z.P., Wu D.C., DiBiase A., Martin C.S., Cech J.N., Sessa A.K., Leblanc J.L. (2011). Lineage regulators direct BMP and Wnt pathways to cell-specific programs during differentiation and regeneration. Cell.

[58] Boria I., Garelli E., Gazda H.T., Aspesi A., Quarello P., Pavesi E., Ferrante D., Meerpohl J.J., Kartal M., Da Costa L. (2010). The ribosomal basis of Diamond-Blackfan Anemia: Mutation and database update. Hum. Mutat..

[59] Ludwig L.S., Gazda H.T., Eng J.C., Eichhorn S.W., Thiru P., Ghazvinian R., George T.I., Gotlib J.R., Beggs A.H., Sieff C.A. (2014). Altered translation of GATA1 in Diamond-Blackfan anemia. Nat. Med..

[60] Hentze M.W., Kuhn L.C. (1996). Molecular control of vertebrate iron metabolism: mRNA-based regulatory circuits operated by iron, nitric oxide, and oxidative stress. Proc. Natl. Acad. Sci. USA.

[61] Fibach E., Manor D., Oppenheim A., Rachmilewitz E.A. (1989). Proliferation and maturation of human erythroid progenitors in liquid culture. Blood.

[62] Wada H., Suda T., Miura Y., Kajii E., Ikemoto S., Yawata Y. (1990). Expression of major blood group antigens on human erythroid cells in a two phase liquid culture system. Blood.

[63] Fibach E., Rachmilewitz E.A. (1991). A two-step liquid culture—A novel culture procedure for studying erythroid cell development. Haematologia.

[64] Wright D.E., Wagers A.J., Gulati A.P., Johnson F.L., Weissman I.L. (2001). Physiological migration of hematopoietic stem and progenitor cells. Science.

[65] Massberg S., Schaerli P., Knezevic-Maramica I., Kollnberger M., Tubo N., Moseman E.A., Huff I.V., Junt T., Wagers A.J., Mazo I.B. (2007). Immunosurveillance by hematopoietic progenitor cells trafficking through blood, lymph, and peripheral tissues. Cell.

[66] Singh V.K., Saini A., Tsuji K., Sharma P.B., Chandra R. (2014). Manufacturing blood ex vivo: A futuristic approach to deal with the supply and safety concerns. Front. Cell. Dev. Biol..

[67] Neildez-Nguyen T.M., Wajcman H., Marden M.C., Bensidhoum M., Moncollin V., Giarratana M.C., Kobari L., Thierry D., Douay L. (2002). Human erythroid cells produced ex vivo at large scale differentiate into red blood cells in vivo. Nat. Biotechnol..

[68] Giarratana M.C., Kobari L., Lapillonne H., Chalmers D., Kiger L., Cynober T., Marden M.C., Wajcman H., Douay L. (2005). Ex vivo generation of fully mature human red blood cells from hematopoietic stem cells. Nat. Biotechnol..

[69] Miharada K., Hiroyama T., Sudo K., Nagasawa T., Nakamura Y. (2006). Efficient enucleation of erythroblasts differentiated in vitro from hematopoietic stem and progenitor cells. Nat. Biotechnol..

[70] Ohneda O., Bautch V.L. (1997). Murine endothelial cells support fetal liver erythropoiesis and myelopoiesis via distinct interactions. Br. J. Haematol..

[71] Hanspal M., Smockova Y., Uong Q. (1998). Molecular identification and functional characterization of a novel protein that mediates the attachment of erythroblasts to macrophages. Blood.

[72] Chasis J.A., Mohandas N. (2008). Erythroblastic islands: Niches for erythropoiesis. Blood.

[73] Fujimi A., Matsunaga T., Kobune M., Kawano Y., Nagaya T., Tanaka I., Iyama S., Hayashi T., Sato T., Miyanishi K. (2008). Ex vivo large-scale generation of human red blood cells from cord blood CD34+ cells by co-culturing with macrophages. Int. J. Hematol..

[74] Hanspal M., Hanspal J.S. (1994). The association of erythroblasts with macrophages promotes erythroid proliferation and maturation: A 30-kD heparin-binding protein is involved in this contact. Blood.

[75] Iavarone A., King E.R., Dai X.M., Leone G., Stanley E.R., Lasorella A. (2004). Retinoblastoma promotes definitive erythropoiesis by repressing Id2 in fetal liver macrophages. Nature.

[76] Webb S. (2013). Banking on cord blood stem cells. Nat. Biotechnol..

[77] Olivier E.N., Qiu C., Velho M., Hirsch R.E., Bouhassira E.E. (2006). Large-scale production of embryonic red blood cells from human embryonic stem cells. Exp. Hematol..

[78] Ma F., Wang D., Hanada S., Ebihara Y., Kawasaki H., Zaike Y., Heike T., Nakahata T., Tsuji K. (2007). Novel method for efficient production of multipotential hematopoietic progenitors from human embryonic stem cells. Int. J. Hematol..

[79] Ma F., Ebihara Y., Umeda K., Sakai H., Hanada S., Zhang H., Zaike Y., Tsuchida E., Nakahata T., Nakauchi H. (2008). Generation of functional erythrocytes from human embryonic stem cell-derived definitive hematopoiesis. Proc. Natl. Acad. Sci. USA.

[80] Dias J., Gumenyuk M., Kang H., Vodyanik M., Yu J., Thomson J.A., Slukvin I.I. (2011). Generation of red blood cells from human induced pluripotent stem cells. Stem Cells Dev..

[81] Haro-Mora J.J., Uchida N., Demirci S., Wang Q., Zou J., Tisdale J.F. (2020). Biallelic correction of sickle cell disease-derived induced pluripotent stem cells (iPSCs) confirmed at the protein level through serum-free iPS-sac/erythroid differentiation. Stem Cells Transl. Med..

[82] Uchida N., Haro-Mora J.J., Fujita A., Lee D.Y., Winkler T., Hsieh M.M., Tisdale J.F. (2017). Efficient Generation of beta-Globin-Expressing Erythroid Cells Using Stromal Cell-Derived Induced Pluripotent Stem Cells from Patients with Sickle Cell Disease. Stem Cells.

[83] Cerdan C., Rouleau A., Bhatia M. (2004). VEGF-A165 augments erythropoietic development from human embryonic stem cells. Blood.

[84] Chang K.H., Nelson A.M., Cao H., Wang L., Nakamoto B., Ware C.B., Papayannopoulou T. (2006). Definitive-like erythroid cells derived from human embryonic stem cells coexpress high levels of embryonic and fetal globins with little or no adult globin. Blood.

[85] Lu S.J., Feng Q., Park J.S., Vida L., Lee B.S., Strausbauch M., Wettstein P.J., Honig G.R., Lanza R. (2008). Biologic properties and enucleation of red blood cells from human embryonic stem cells. Blood.

[86] Lapillonne H., Kobari L., Mazurier C., Tropel P., Giarratana M.C., Zanella-Cleon I., Kiger L., Wattenhofer-Donze M., Puccio H., Hebert N. (2010). Red blood cell generation from human induced pluripotent stem cells: Perspectives for transfusion medicine. Haematologica.

[87] Dorn I., Klich K., Arauzo-Bravo M.J., Radstaak M., Santourlidis S., Ghanjati F., Radke T.F., Psathaki O.E., Hargus G., Kramer J. (2015). Erythroid differentiation of human induced pluripotent stem cells is independent of donor cell type of origin. Haematologica.

[88] Olivier E.N., Marenah L., McCahill A., Condie A., Cowan S., Mountford J.C. (2016). High-Efficiency Serum-Free Feeder-Free Erythroid Differentiation of Human Pluripotent Stem Cells Using Small Molecules. Stem Cells Transl. Med..

[89] Kessel K.U., Bluemke A., Scholer H.R., Zaehres H., Schlenke P., Dorn I. (2017). Emergence of CD43-Expressing Hematopoietic Progenitors from Human Induced Pluripotent Stem Cells. Transfus. Med. Hemother..

[90] Bernecker C., Ackermann M., Lachmann N., Rohrhofer L., Zaehres H., Arauzo-Bravo M.J., van den Akker E., Schlenke P., Dorn I. (2019). Enhanced Ex Vivo Generation of Erythroid Cells from Human Induced Pluripotent Stem Cells in a Simplified Cell Culture System with Low Cytokine Support. Stem Cells Dev..

[91] Roh J., Kim S., Cheong J.W., Jeon S.H., Kim H.K., Kim M.J., Kim H.O. (2022). Erythroid Differentiation of Induced Pluripotent Stem Cells Co-cultured with OP9 Cells for Diagnostic Purposes. Ann. Lab. Med..

[92] Smith B.W., Rozelle S.S., Leung A., Ubellacker J., Parks A., Nah S.K., French D., Gadue P., Monti S., Chui D.H. (2013). The aryl hydrocarbon receptor directs hematopoietic progenitor cell expansion and differentiation. Blood.

[93] Olivier E.N., Zhang S., Yan Z., Suzuka S., Roberts K., Wang K., Bouhassira E.E. (2019). PSC-RED and MNC-RED: Albumin-free and low-transferrin robust erythroid differentiation protocols to produce human enucleated red blood cells. Exp. Hematol..

[94] Poldee S., Metheetrairut C., Nugoolsuksiri S., Frayne J., Trakarnsanga K. (2018). Optimization of an erythroid culture system to reduce the cost of in vitro production of red blood cells. MethodsX.

[95] Netsrithong R., Suwanpitak S., Boonkaew B., Trakarnsanga K., Chang L.J., Tipgomut C., Vatanashevanopakorn C., Pattanapanyasat K., Wattanapanitch M. (2020). Multilineage differentiation potential of hematoendothelial progenitors derived from human induced pluripotent stem cells. Stem Cell Res. Ther..

[96] Niwa A., Heike T., Umeda K., Oshima K., Kato I., Sakai H., Suemori H., Nakahata T., Saito M.K. (2011). A novel serum-free monolayer culture for orderly hematopoietic differentiation of human pluripotent cells via mesodermal progenitors. PLoS ONE.

[97] Chou S.T., Byrska-Bishop M., Tober J.M., Yao Y., Vandorn D., Opalinska J.B., Mills J.A., Choi J.K., Speck N.A., Gadue P. (2012). Trisomy 21-associated defects in human primitive hematopoiesis revealed through induced pluripotent stem cells. Proc. Natl. Acad. Sci. USA.

[98] Tursky M.L., Loi T.H., Artuz C.M., Alateeq S., Wolvetang E.J., Tao H., Ma D.D., Molloy T.J. (2020). Direct Comparison of Four Hematopoietic Differentiation Methods from Human Induced Pluripotent Stem Cells. Stem Cell Rep..

[99] Leary A.G., Ikebuchi K., Hirai Y., Wong G.G., Yang Y.C., Clark S.C., Ogawa M. (1988). Synergism between interleukin-6 and interleukin-3 in supporting proliferation of human hematopoietic stem cells: Comparison with interleukin-1 alpha. Blood.

[100] Ulich T.R., del Castillo J., Yin S.M., Egrie J.C. (1991). The erythropoietic effects of interleukin 6 and erythropoietin in vivo. Exp. Hematol..

[101] Kieran M.W., Perkins A.C., Orkin S.H., Zon L.I. (1996). Thrombopoietin rescues in vitro erythroid colony formation from mouse embryos lacking the erythropoietin receptor. Proc. Natl. Acad. Sci. USA.

[102] Wu H., Klingmuller U., Acurio A., Hsiao J.G., Lodish H.F. (1997). Functional interaction of erythropoietin and stem cell factor receptors is essential for erythroid colony formation. Proc. Natl. Acad. Sci. USA.

[103] Lyman S.D., Jacobsen S.E. (1998). c-kit ligand and Flt3 ligand: Stem/progenitor cell factors with overlapping yet distinct activities. Blood.

[104] Wannatung T., Lithanatudom P., Leecharoenkiat A., Svasti S., Fucharoen S., Smith D.R. (2009). Increased erythropoiesis of beta-thalassaemia/Hb E proerythroblasts is mediated by high basal levels of ERK1/2 activation. Br. J. Haematol..

[105] Diaz M.F., Li N., Lee H.J., Adamo L., Evans S.M., Willey H.E., Arora N., Torisawa Y.S., Vickers D.A., Morris S.A. (2015). Biomechanical forces promote blood development through prostaglandin E2 and the cAMP-PKA signaling axis. J. Exp. Med..

[106] Bratt-Leal A.M., Carpenedo R.L., McDevitt T.C. (2009). Engineering the embryoid body microenvironment to direct embryonic stem cell differentiation. Biotechnol. Prog..

[107] McGrath K.E., Frame J.M., Fromm G.J., Koniski A.D., Kingsley P.D., Little J., Bulger M., Palis J. (2011). A transient definitive erythroid lineage with unique regulation of the beta-globin locus in the mammalian embryo. Blood.

[108] Friedman J.M. (1985). Structure, dynamics, and reactivity in hemoglobin. Science.

[109] Gell D.A. (2018). Structure and function of haemoglobins. Blood Cells Mol. Dis..

[110] Wood W.G., Weatherall D.J. (1973). Haemoglobin synthesis during human foetal development. Nature.

[111] Qiu C., Hanson E., Olivier E., Inada M., Kaufman D.S., Gupta S., Bouhassira E.E. (2005). Differentiation of human embryonic stem cells into hematopoietic cells by coculture with human fetal liver cells recapitulates the globin switch that occurs early in development. Exp. Hematol..

[112] Umeda K., Heike T., Nakata-Hizume M., Niwa A., Arai M., Shinoda G., Ma F., Suemori H., Luo H.Y., Chui D.H. (2006). Sequential analysis of alpha- and beta-globin gene expression during erythropoietic differentiation from primate embryonic stem cells. Stem Cells.

[113] Peschle C., Mavilio F., Care A., Migliaccio G., Migliaccio A.R., Salvo G., Samoggia P., Petti S., Guerriero R., Marinucci M. (1985). Haemoglobin switching in human embryos: Asynchrony of zeta → alpha and epsilon → gamma-globin switches in primitive and definite erythropoietic lineage. Nature.

[114] Qiu C., Olivier E.N., Velho M., Bouhassira E.E. (2008). Globin switches in yolk sac-like primitive and fetal-like definitive red blood cells produced from human embryonic stem cells. Blood.

[115] Ochi K., Takayama N., Hirose S., Nakahata T., Nakauchi H., Eto K. (2014). Multicolor staining of globin subtypes reveals impaired globin switching during erythropoiesis in human pluripotent stem cells. Stem Cells Transl. Med..

[116] Pevny L., Simon M.C., Robertson E., Klein W.H., Tsai S.F., D’Agati V., Orkin S.H., Costantini F. (1991). Erythroid differentiation in chimaeric mice blocked by a targeted mutation in the gene for transcription factor GATA-1. Nature.

[117] Basu P., Lung T.K., Lemsaddek W., Sargent T.G., Williams D.C., Basu M., Redmond L.C., Lingrel J.B., Haar J.L., Lloyd J.A. (2007). EKLF and KLF2 have compensatory roles in embryonic beta-globin gene expression and primitive erythropoiesis. Blood.

[118] Yang C.T., Ma R., Axton R.A., Jackson M., Taylor A.H., Fidanza A., Marenah L., Frayne J., Mountford J.C., Forrester L.M. (2017). Activation of KLF1 Enhances the Differentiation and Maturation of Red Blood Cells from Human Pluripotent Stem Cells. Stem Cells.

[119] Song S.H., Hou C., Dean A. (2007). A positive role for NLI/Ldb1 in long-range beta-globin locus control region function. Mol. Cell.

[120] Honig G.R., Lu S.J., Feng Q., Vida L.N., Lee B.S., Lanza R. (2010). Alpha-Thalassemia-like globin gene expression by primitive erythrocytes derived from human embryonic stem cells. Hemoglobin.

[121] Fujita A., Uchida N., Haro-Mora J.J., Winkler T., Tisdale J. (2016). Beta-Globin-Expressing Definitive Erythroid Progenitor Cells Generated from Embryonic and Induced Pluripotent Stem Cell-Derived Sacs. Stem Cells.

[122] Sassa S., Nagai T. (1996). The role of heme in gene expression. Int. J. Hematol..

[123] Chan K.S., Xu J., Wardan H., McColl B., Orkin S., Vadolas J. (2012). Generation of a genomic reporter assay system for analysis of gamma- and beta-globin gene regulation. FASEB J..

[124] Suzuki M., Yamamoto M., Engel J.D. (2014). Fetal globin gene repressors as drug targets for molecular therapies to treat the beta-globinopathies. Mol. Cell. Biol..

[125] Yu L., Myers G., Engel J.D. (2020). Small molecule therapeutics to treat the beta-globinopathies. Curr. Opin. Hematol..

[126] Soboleva S., Kurita R., Ek F., Akerstrand H., Silverio-Alves R., Olsson R., Nakamura Y., Miharada K. (2021). Identification of potential chemical compounds enhancing generation of enucleated cells from immortalized human erythroid cell lines. Commun. Biol..

[127] Xu J., Sankaran V.G., Ni M., Menne T.F., Puram R.V., Kim W., Orkin S.H. (2010). Transcriptional silencing of gamma-globin by BCL11A involves long-range interactions and cooperation with SOX6. Genes Dev..

[128] Trakarnsanga K., Wilson M.C., Lau W., Singleton B.K., Parsons S.F., Sakuntanaga P., Kurita R., Nakamura Y., Anstee D.J., Frayne J. (2014). Induction of adult levels of beta-globin in human erythroid cells that intrinsically express embryonic or fetal globin by transduction with KLF1 and BCL11A-XL. Haematologica.

[129] Bauer D.E., Orkin S.H. (2015). Hemoglobin switching’s surprise: The versatile transcription factor BCL11A is a master repressor of fetal hemoglobin. Curr. Opin. Genet. Dev..

[130] Liu N., Hargreaves V.V., Zhu Q., Kurland J.V., Hong J., Kim W., Sher F., Macias-Trevino C., Rogers J.M., Kurita R. (2018). Direct Promoter Repression by BCL11A Controls the Fetal to Adult Hemoglobin Switch. Cell.

[131] Chang K.H., Huang A., Hirata R.K., Wang P.R., Russell D.W., Papayannopoulou T. (2010). Globin phenotype of erythroid cells derived from human induced pluripotent stem cells. Blood.

[132] Ng E.S., Azzola L., Bruveris F.F., Calvanese V., Phipson B., Vlahos K., Hirst C., Jokubaitis V.J., Yu Q.C., Maksimovic J. (2016). Differentiation of human embryonic stem cells to HOXA^+^ hemogenic vasculature that resembles the aorta-gonad-mesonephros. Nat. Biotechnol..

[133] Razaq M.A., Taylor S., Roberts D.J., Carpenter L. (2017). A molecular roadmap of definitive erythropoiesis from human induced pluripotent stem cells. Br. J. Haematol..

[134] Vanuytsel K., Matte T., Leung A., Naing Z.H., Morrison T., Chui D.H.K., Steinberg M.H., Murphy G.J. (2018). Induced pluripotent stem cell-based mapping of beta-globin expression throughout human erythropoietic development. Blood Adv..

[135] Trakarnsanga K., Wilson M.C., Heesom K.J., Andrienko T.N., Srisawat C., Frayne J. (2018). Secretory factors from OP9 stromal cells delay differentiation and increase the expansion potential of adult erythroid cells in vitro. Sci. Rep..

[136] Zhou D., Liu K., Sun C.W., Pawlik K.M., Townes T.M. (2010). KLF1 regulates BCL11A expression and gamma- to beta-globin gene switching. Nat. Genet..

[137] Hanna J., Wernig M., Markoulaki S., Sun C.W., Meissner A., Cassady J.P., Beard C., Brambrink T., Wu L.C., Townes T.M. (2007). Treatment of sickle cell anemia mouse model with iPS cells generated from autologous skin. Science.

[138] Abboud S., Haile D.J. (2000). A novel mammalian iron-regulated protein involved in intracellular iron metabolism. J. Biol. Chem..

[139] Nemeth E., Tuttle M.S., Powelson J., Vaughn M.B., Donovan A., Ward D.M., Ganz T., Kaplan J. (2004). Hepcidin regulates cellular iron efflux by binding to ferroportin and inducing its internalization. Science.

[140] Rouault T.A. (2006). The role of iron regulatory proteins in mammalian iron homeostasis and disease. Nat. Chem. Biol..

[141] Crooks D.R., Ghosh M.C., Haller R.G., Tong W.H., Rouault T.A. (2010). Posttranslational stability of the heme biosynthetic enzyme ferrochelatase is dependent on iron availability and intact iron-sulfur cluster assembly machinery. Blood.

[142] Ghosh M.C., Zhang D.L., Jeong S.Y., Kovtunovych G., Ollivierre-Wilson H., Noguchi A., Tu T., Senecal T., Robinson G., Crooks D.R. (2013). Deletion of iron regulatory protein 1 causes polycythemia and pulmonary hypertension in mice through translational derepression of HIF2alpha. Cell Metab..

[143] Wilkinson N., Pantopoulos K. (2013). IRP1 regulates erythropoiesis and systemic iron homeostasis by controlling HIF2alpha mRNA translation. Blood.

[144] Zhang D.L., Ghosh M.C., Rouault T.A. (2014). The physiological functions of iron regulatory proteins in iron homeostasis—An update. Front. Pharmacol..

[145] Meyron-Holtz E.G., Ghosh M.C., Iwai K., LaVaute T., Brazzolotto X., Berger U.V., Land W., Ollivierre-Wilson H., Grinberg A., Love P. (2004). Genetic ablations of iron regulatory proteins 1 and 2 reveal why iron regulatory protein 2 dominates iron homeostasis. EMBO J..

[146] Smith S.R., Ghosh M.C., Ollivierre-Wilson H., Hang Tong W., Rouault T.A. (2006). Complete loss of iron regulatory proteins 1 and 2 prevents viability of murine zygotes beyond the blastocyst stage of embryonic development. Blood Cells Mol. Dis..

[147] Cooperman S.S., Meyron-Holtz E.G., Olivierre-Wilson H., Ghosh M.C., McConnell J.P., Rouault T.A. (2005). Microcytic anemia, erythropoietic protoporphyria, and neurodegeneration in mice with targeted deletion of iron-regulatory protein 2. Blood.

[148] Soboleva S., Miharada K. (2022). Induction of enucleation in primary and immortalized erythroid cells. Int. J. Hematol..

[149] Kawabata H. (2019). Transferrin and transferrin receptors update. Free Radic. Biol. Med..

[150] Canonne-Hergaux F., Zhang A.S., Ponka P., Gros P. (2001). Characterization of the iron transporter DMT1 (NRAMP2/DCT1) in red blood cells of normal and anemic mk/mk mice. Blood.

[151] Meng F., Fleming B.A., Jia X., Rousek A.A., Mulvey M.A., Ward D.M. (2022). Lysosomal iron recycling in mouse macrophages is dependent upon both LcytB and Steap3 reductases. Blood Adv..

[152] Kennedy M., Firpo M., Choi K., Wall C., Robertson S., Kabrun N., Keller G. (1997). A common precursor for primitive erythropoiesis and definitive haematopoiesis. Nature.

[153] McGrath K.E., Kingsley P.D., Koniski A.D., Porter R.L., Bushnell T.P., Palis J. (2008). Enucleation of primitive erythroid cells generates a transient population of “pyrenocytes” in the mammalian fetus. Blood.

[154] Ebrahimi M., Forouzesh M., Raoufi S., Ramazii M., Ghaedrahmati F., Farzaneh M. (2020). Differentiation of human induced pluripotent stem cells into erythroid cells. Stem Cell. Res. Ther..

[155] Moras M., Lefevre S.D., Ostuni M.A. (2017). From Erythroblasts to Mature Red Blood Cells: Organelle Clearance in Mammals. Front. Physiol..

[156] Simpson C.F., Kling J.M. (1967). The mechanism of denucleation in circulating erythroblasts. J. Cell. Biol..

[157] Tallack M.R., Keys J.R., Humbert P.O., Perkins A.C. (2009). EKLF/KLF1 controls cell cycle entry via direct regulation of E2f2. J. Biol. Chem..

[158] Gnanapragasam M.N., McGrath K.E., Catherman S., Xue L., Palis J., Bieker J.J. (2016). EKLF/KLF1-regulated cell cycle exit is essential for erythroblast enucleation. Blood.

[159] Swartz K.L., Wood S.N., Murthy T., Ramirez O., Qin G., Pillai M.M., Rao S., Minella A.C. (2017). E2F-2 Promotes Nuclear Condensation and Enucleation of Terminally Differentiated Erythroblasts. Mol. Cell. Biol..

[160] Jayapal S.R., Lee K.L., Ji P., Kaldis P., Lim B., Lodish H.F. (2010). Down-regulation of Myc is essential for terminal erythroid maturation. J. Biol. Chem..

[161] Zhao B., Mei Y., Schipma M.J., Roth E.W., Bleher R., Rappoport J.Z., Wickrema A., Yang J., Ji P. (2016). Nuclear Condensation during Mouse Erythropoiesis Requires Caspase-3-Mediated Nuclear Opening. Dev. Cell.

[162] Ji P., Yeh V., Ramirez T., Murata-Hori M., Lodish H.F. (2010). Histone deacetylase 2 is required for chromatin condensation and subsequent enucleation of cultured mouse fetal erythroblasts. Haematologica.

[163] Zhang L., Flygare J., Wong P., Lim B., Lodish H.F. (2011). miR-191 regulates mouse erythroblast enucleation by down-regulating Riok3 and Mxi1. Genes Dev..

[164] Ji P., Jayapal S.R., Lodish H.F. (2008). Enucleation of cultured mouse fetal erythroblasts requires Rac GTPases and mDia2. Nat. Cell. Biol..

[165] Keerthivasan G., Small S., Liu H., Wickrema A., Crispino J.D. (2010). Vesicle trafficking plays a novel role in erythroblast enucleation. Blood.

[166] Keerthivasan G., Liu H., Gump J.M., Dowdy S.F., Wickrema A., Crispino J.D. (2012). A novel role for survivin in erythroblast enucleation. Haematologica.

[167] Timmins N.E., Athanasas S., Gunther M., Buntine P., Nielsen L.K. (2011). Ultra-high-yield manufacture of red blood cells from hematopoietic stem cells. Tissue Eng. C Methods.

[168] Bernecker C., Kofeler H., Pabst G., Trotzmuller M., Kolb D., Strohmayer K., Trajanoski S., Holzapfel G.A., Schlenke P., Dorn I. (2019). Cholesterol Deficiency Causes Impaired Osmotic Stability of Cultured Red Blood Cells. Front. Physiol..

